# An unusual case of frost bite autoamputation of toes

**DOI:** 10.1186/1757-1626-1-398

**Published:** 2008-12-16

**Authors:** Adil Hafeez Wani, Mir Mohsin, Mohammed Ashraf Darzi, Mohammed Inam Zaroo, Sheikh Adil Bashir, Haroon Rashid Zargar, Altaf Rasool, Mohammed Akram Bijli, Hameedullah Dar, Peerzada Omar Farooq, Sheikh Tariq Ahmed

**Affiliations:** 1 Department of Plastic and Reconstructive Surgery, Sheri Kashmir Institute of Medical Sciences, Srinagar, Kashmir, Jammu and Kashmi, India

## Abstract

**Background:**

We report a case of a 15 year old young female who suffered autoamputation of left mid foot and four digits of right foot following repeated application of snow to relieve the pain in her frost bitten feet.

**Case presentation:**

The sociodemographic background, cause, resulting injury and subsequent management are discussed.

**Conclusion:**

Such injuries are relatively rare but awareness of the risk of this type of injury is important.

## Background

Injury due to cold may be general or local. Local cold injury may occur at temperatures above freezing (wet-cold conditions), as in immersion or trench foot. At temperatures below freezing (dry-cold conditions), frost bite occurs; the tissues freeze and ice crystals form in between the cells[1]. Local cold injury may or may not be associated with hypothermia. Although frost bite is the most common cold injury[2], in civilian life, frost bite is uncommon despite populations of about 100 million at risk in areas where sub-zero temperatures occur at some period of the year[1]. Among mountaineers at high altitude cases still occur regularly[2].

Maintenance of the central core temperature is essential to life and this may be carried out at the expense of the peripheral expendable structures such as the toes and fingers[1]. Cold damages tissues through cellular injury and vascular impairment[3]. Cellular injury may be due to intracellular water crystallization, temperature-induced protein changes and membrane damage[4]. Vasoconstriction, endothelial injury and thromboembolism contribute to vascular insufficiency and ischemia. Overtime, necrosis and gangrene becomes apparent[2]. Mummification and autoamputation may occur.[5]

Because of the in affordability of the expenses involved in the traditional marriages with regional women, a trend of marrying women from outside the state usually belonging to Bihar and West Bengal has developed of late among the men of Kashmir valley, India. Though such marriages involve least expenses as most of the costly traditions are by passed but the new bride takes a lot of time to adjust to the new climate and culture, at times leading to unfortunate accidents because of lack of proper knowledge to work their way out in different weathers.

## Case presentation

Socio-demographic background: The patient originally a non-Kashmiri belonged to West Bengal, a state in Eastern India where the temperature even in winter doesn't drop below 20°C. She was married to a villager of low socioeconomic status from Kashmir 2 years back.

**Figure 1:**
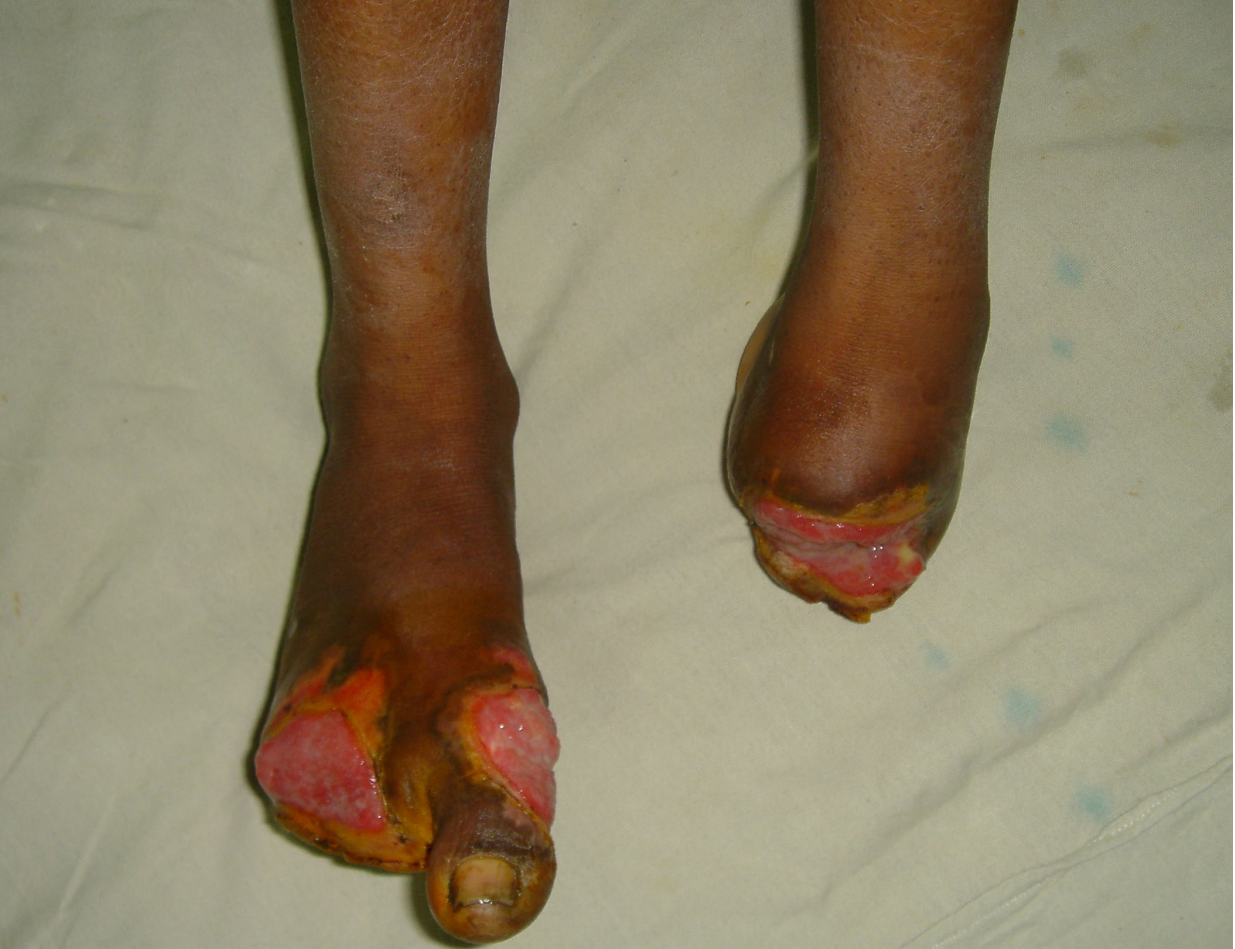
Amputated stump left mid foot with an ulcer and amputated lateral four toes of right foot with infected wounds over the stump.

The patient, a young female of low socioeconomic status presently resident of a mountainous region, had suffered frostbite to her feet while dwelling outdoors in snow to collect water and timber for her household. She devised her own way of getting relief from the pain by applying snow on her feet repeatedly till they became insensate and turned black. She reported in the Out patient department (OPD) of the Department of Plastic and Reconstructive Surgery of Sheri-Kashmir Institute of Medical Sciences, Srinagar, after 15 days with autoamputated left mid foot and the lateral four toes of right foot with infected wounds over the stumps (Figure 1). She was given supportive treatment. Her wounds were managed by VAC (vacuum assisted closure) therapy followed by split thickness skin grafting and patient was discharged with proper advice regarding ways to protect herself in future from cold during winter especially while dwelling outdoors.

## Discussion

Frost bite is the most common cold injury.[2] Extremities are most commonly affected[6]. One of the major groups at risk of frostbite is mountaineers, who are mostly affected in cold seasons and at high altitudes. From the clinical view point it is classified into four degrees of progressive injury identified by physical signs and the following sequelae[7,8]. First degree injury is characterized by epidermal involvement, which causes erythma, mild oedema, and sequelae over the next few weeks such as desquamation and cold sensitivity. Second degree injury is full thickness skin freezing with substantial oedema and formation of clear blisters, which contract and dry within two to three weeks, forming a dark eschar. Third degree injury is characterized by formation of hemorrhagic blisters, blue-grey discolouration of the skin, deep burning pain or rewarming, thick gangrene eschar formation. In fourth degree injury, muscle, bone and tendons are involved. Our patient had probably sustained a fourth degree frost bite injury which had sequentially led to autoamputation of distal portion of her feet over time.

Occurrence of the injury is related to many climatic and personal factors such as low temperature, high altitude, windy weather, tobacco smoking, presence of peripheral vascular disease and behavioural response to cold ambient temperature[2]. Our patient had recently shifted to Kashmir post marriage and unaware of the risks involved and the means to protect herself used snow to relieve her pain due to frostbite thus further aggravating her injury leading to the unfortunate loss of her feet.

The single most important cause of frost bite is inappropriate clothing accounting for 45% of injuries[2]. Our patient a non-Kasmiri had married in a low socio-economic strata of the Kashmir Valley, as such poor clothing, illiteracy, and lack of knowledge about dealing with such problems led to a vicious cycle leading to autoamputation of her feet.

Two main reactions take place when tissues come into contact with a very cold object. Firstly, a vascular reaction occurs under the frozen superficial tissues consisting of damage to the wall of the blood vessels, leakage of plasma into the tissues (forming blisters), and an increased viscosity of the remaining intravascular blood, with local haemoconcentration or "sludging." The small vessels may thus become blocked[9]. If the blood flow is then stopped by the action of the precapillary sphincters, the arteriovenous shunts will open up and blood bypasses the frozen area, which becomes avascular: in other words, the diseased part is sacrificed for survival of the whole organism. The second reaction is the formation of intercellular ice crystals. The intracellular osmotic pressure rises and enzyme mechanisms are disturbed with subsequent cell death[1].

A layering system, which creates a microclimate around the body protecting against cold and wind, is highly efficient in preventing frost bite and hypothermia[2]. Management of such cases demands attention to hypothermia and local cold-induced injury as well as to coexisting trauma, infection and intoxication if any. In the prehospital care of frost bite, nonadherent wet clothing should be removed. Local rewarming started. In hospital, rapid rewarming of a frost bitten extremity in a bath of water between 40°C and 42°C for 15 to 30 minutes may minimize tissue loss[10]. Splinting and elevation of affected part reduce oedema and improve perfusion. Hospital stay for patients with deep frost bite is often prolonged. Surgical amputation may be required many weeks after the injury[2].

The Department of Health and Community Medicine is taking due interest in creating awareness among the public regarding measures to safeguard themselves during winter. Further measures should be taken by the Primary and the Community Health Centers and some NGOs to educate people who migrate to Kashmir (North India) to seek work to earn their livelihood or for other reasons like inter-state marriages, etc, so that such mishappenings are avoided in future.

## Consent

Written informed consent was obtained from the patient for publication of this case report and accompanying image. A copy of the written consent is available for review by the Editor-in-Chief of this journal.

## Competing interests

The authors declare that they have no competing interests.

## Authors' contributions

AHW, MM, MAD, MIZ, SAB, HRZ, AH, MAB, HD made substantial contributions to conception and design, acquisition of data and revising it. POF and STA contributed significantly in acquisition of data and drafting the manuscript.
